# Antimicrobial tele-stewardship strategies and their application to the rural outpatient setting

**DOI:** 10.1017/ash.2025.10133

**Published:** 2025-10-01

**Authors:** Matthew James Peworchik, Ritu Banerjee, Sophie Katz

**Affiliations:** Vanderbilt University Medical Center, Department of Pediatrics, Division of Pediatric Infectious Disease, Nashville, TN, USA

## Abstract

**Objective:**

To review the variety of antimicrobial tele-stewardship strategies and their applicability to the rural outpatient context.

**Design:**

Narrative review of antimicrobial tele-stewardship activities and their potential adaptability to rural outpatient settings.

**Results:**

Few studies evaluate implementation and outcomes of antimicrobial stewardship in rural settings. In this review we summarize the variety of methods that have been used in tele-stewardship implementation research, and how each can be adapted to the rural outpatient setting, including: audit and feedback, peer comparison, provider education, guideline creation, consultation with infectious disease providers, and electronic medical record nudges.

**Conclusions:**

Tele-stewardship activities are likely to benefit outpatient rural settings, though further implementation research is needed.

## Introduction

Rates of antimicrobial-resistant infections are increasing, creating a global public health emergency. In the United States (US) in 2022, six antimicrobial-resistant pathogens including carbapenem-resistant Enterobacterales, vancomycin-resistant *Enterococcus*, and multidrug-resistant *Pseudomonas aeruginosa* increased by a combined 20% compared to prepandemic levels.^1^ One of the most important checks against emergence and spread of resistant pathogens are antimicrobial stewardship programs, which are now widely implemented in a variety of healthcare settings. The Centers for Disease Control and Prevention (CDC) outlines core elements that should be part of an antimicrobial stewardship program in a variety of contexts, including inpatient acute care hospitals, outpatient clinics, long term care facilities, and critical access hospitals.^2,3,4,5^ The CDC’s core elements of outpatient antibiotic stewardship are commitment to optimizing antibiotic prescribing, action for policy and practice, tracking and reporting, and education and expertise (Table 1).^6^ However, there are no guidelines that specifically address how to implement antibiotic stewardship programs in rural outpatient settings.

**Table 1.** CDC core elements for outpatient stewardship and associated applicable tele-stewardship interventions

CDC Core element	Applicable Tele-stewardship Interventions
Commitment to optimizing antibiotic prescribing	Commitment statements from practices that were involved, posters in involved practices showing commitment to antibiotic stewardship
Action for policy and practice	Dissemination of antibiotic use reports with peer comparison, creation of antibiotic use guidelines, making infectious disease consultation more widely available via telemedicine, changes to default antibiotic courses in the EHR
Tracking and Reporting	Dissemination of antibiotic use reports with peer comparison
Education and Expertise	Educational sessions for providers, making infectious disease consultation more widely available via telemedicine

Rural areas have a variety of unique healthcare challenges including less access to hospital and subspecialist care and long distances to primary care provider offices. This lack of subspecialist care seen in rural areas includes infectious disease (ID) subspecialists.^7^ Rural areas have higher rates of chronic disease and worse healthcare outcomes compared to urban settings.^8^ A variety of studies have shown higher rates of antimicrobial prescription in rural areas. Over a 6-year period among Kentucky children with Medicaid, those who lived in a rural setting received a median of 9 antibiotic prescriptions per year compared to 4 prescriptions per year in children that lived in an urban setting.^9^ Another study on children with Medicaid, this time in Tennessee, found that children living in mostly rural or completely rural counties had higher rates of inappropriate antibiotic prescriptions for acute respiratory infections compared to mostly urban counties (adjusted incidence rate ratio 1.34 and 1.33).^10^ A third study found that adults who saw their primary care provider for an upper respiratory infection (URI) in a rural setting were more likely to be prescribed an unnecessary antibiotic than those in an urban setting (OR 1.49).^11^

Implementing antimicrobial stewardship interventions in the rural outpatient context poses unique challenges that have not been well studied. One implementation strategy that has recently gained greater attention is tele-stewardship, which involves antimicrobial stewardship interventions delivered via telecommunication and electronic information technology using off-site resources. Recent reviews suggest these interventions often result in overall lower antibiotic use. Mailig et al reviewed 14 tele-stewardship interventions and found that 60% showed an overall decrease in antimicrobial use, a third of which were statistically significant.^12^ Pierce and Stevens found that 4 of 5 tele-stewardship studies reported significant reductions in antimicrobial use.^13^ In this review, we summarize recent data about implementation and outcomes of tele-stewardship and examine how they might be applied to outpatient rural settings (Table 2).

**Table 2.** Characteristics of recent telehealth implementation studies

Study	Setting	Adult/Pediatric	Strategies used
Stenehjem, 2023^5^	Outpatient (Urgent Cares in an integrated healthcare system)	Both	Patient education, prescriber education, EHR nudges, audit and feedback with peer comparison
Beaulac, 2016^2^	Inpatient (one long term acute care hospital)	Adult	Antibiotic use reports, audit and feedback
Cantey, 2022^16^	Inpatient (level 1 nurseries)	Pediatric	Provider education, audit and feedback with peer comparison, consultation with ID providers
Jelinski, 2023^26^	Inpatient (hospitals and long term care facilities)	Both	Provider education, consultation with ID providers
Klatt, 2021^4^	Inpatient (community hospital)	Adult	Audit and feedback, guideline creation, EHR nudges, provider education
May, 2023^18^	Inpatient (community hospitals)	Adult	Audit and feedback
Shively, 2023^17^	Inpatient (community hospitals)	Adult	Audit and feedback, provider education, EHR nudges
Stenehjem, 2018^20^	Inpatient (small community hospitals)	Adult	Provider education, antimicrobial use reports, audit and feedback, EHR nudges
Moehring, 2021^22^	Inpatient (community hospitals)	Adult	EHR nudges, provider education, guideline creation
Wilson, 2019^19^	Inpatient (2 rural Veterans Affairs medical centers, both acute and long term care)	Adult	Audit and feedback
Gerber, 2013^14^	Outpatient (Hospital network associated Primary Cares, both academic and community)	Pediatric	Provider education, audit and feedback with peer comparison
Cummings, 2020^21^	Outpatient (Urgent Care centers)	Adult	Patient education, provider education, audit and feedback with peer comparison

## Methods

A PubMed search for articles including both “telehealth” or “telemedicine” and “stewardship”, “antibiotic stewardship”, or “antimicrobial stewardship” was performed to identify relevant articles. No time frame was applied to the search as most research on these topics is recent. Results were then examined for studies implementing a tele-stewardship intervention or reviewing the results of multiple such interventions. Some relevant articles already known to the authors but not present in the PubMed search were included as well.^11,14,15,16^

## Strategies

To accomplish the goals of CDC’s Core Elements, a variety of tele-stewardship interventions have been evaluated including audit and feedback about antibiotic prescribing, peer comparison, provider education, direct consultations with patients and prescribers, practice guideline development, and electronic health record (EHR) modifications designed to improve antibiotic use. These all are applicable to the rural outpatient setting.

### Audit and feedback

The most widely used strategy for tele-stewardship programs has been audit and feedback of antibiotic prescribing.^13^ This intervention consists of tracking the amount and appropriateness of antibiotic use at a clinic or provider level and offering feedback to the prescriber regarding alternative courses of action if indicated.^17^ Audit and feedback can take multiple forms, including prospective audit and feedback that intervenes directly on new antimicrobial courses as they are prescribed as well as retrospective approaches that intermittently provide prescribers with feedback on their overall prescribing practices. This strategy has been used extensively in the inpatient setting where it is usually performed either by a pharmacist or ID physician.^12^ Audit and feedback has also been used successfully in outpatient settings. In a study by Gerber et al, quarterly audit and feedback sessions were performed with providers at selected pediatric primary care offices in non-rural parts of Pennsylvania and New Jersey, resulting in significant decreases in antibiotic prescribing compared to control sites where no intervention was performed.^14^ A follow-up study by the same group showed that once the audit and feedback intervention was removed, antibiotic prescribing increased back to baseline levels.^15^ Audit and feedback interventions align with many of the core elements outlined by the CDC, as they involve action for practice, tracking/reporting prescribing, leveraging antimicrobial prescribing expertise, and educating prescribers. This practice is often the core of a tele-stewardship program, has been effective at reducing antibiotic use, and has been implemented in some studies as the sole intervention.^18,19^ The specific methodology of audit and feedback varies to some degree and ranges from giving direct feedback on patients’ ongoing antimicrobial therapy (more common in inpatient implementation) to more intermittent feedback on prescribing trends (more common for outpatient). Studies examining audit and feedback as a sole intervention were often more limited in scope; one study was specific to decreasing antimicrobial prescriptions for COVID-19 pneumonia,^18^ though it did show decreased overall antibiotic use in the specific context studied. Another study that effectively demonstrated the efficacy of audit and feedback was conducted by Stenehjem et al using three sets of stewardship interventions of increasing intensity in 15 small hospitals in the Intermountain healthcare system. The first set included basic stewardship education, an ID hotline, and an antimicrobial use report. The second set of interventions added advanced stewardship education, local antibiotic restriction, and limited prospective audit and feedback involving pharmacist review of a few specific antibiotics (vancomycin, piperacillin/tazobactam, cefepime, and carbapenems) for opportunities for de-escalation, bug/drug mismatches, and indications for contacting an ID clinician. The third set expanded these interventions to include ID clinician approval for restricted antibiotics, ID clinician review of designated cultures, and expanded the audit and feedback initiated in the second set of interventions to a broader variety of antibiotics ordered for inpatients. Only the third set of interventions including the most intensive stewardship interventions like expanded audit and feedback was associated with a significant reduction in aggregate antimicrobial use.^20^ These results highlight the importance of audit and feedback as a component of a successful stewardship program.

The advantages of audit and feedback are likely as applicable to the rural outpatient context as they are to the inpatient setting. However, there are some features of rural outpatient settings that make audit and feedback more difficult to implement. As outpatient visits are brief and further contact with patients after the initial visit does not always occur, any prospective audit and feedback on ongoing courses of antimicrobials as is often done in inpatient programs will be difficult. An additional challenge making prospective audit and feedback impractical is the higher volume of patients seen in the outpatient setting compared to inpatient. The sort of personalized feedback required for this intervention is resource-intensive and impractical to implement across an outpatient healthcare network. These disadvantages would likely be more pronounced in rural settings where there are fewer providers available leading to higher individual patient volumes and less access to support from larger health systems. Instead, studies focused on tele-stewardship in the outpatient context tend to rely on more asynchronous methods of stewardship, such as prescriber education or distribution of antibiotic use reports, that do not require real-time clinical decision support from stewardship personnel.^5^

### Antibiotic use reports

Antibiotic use reports are a less labor-intensive version of the audit and feedback intervention. Such interventions summarize antibiotic use in a particular context over time and often benchmark antibiotic use against other sites or national averages. These reports can also be individualized to the provider level. Antibiotic use reports have been included as a component of a variety of tele-stewardship studies based in diverse settings including urgent cares, community hospitals, and long term care hospitals.^20^ These studies showed improvement in some measures of antimicrobial prescribing, but used bundled interventions, so the degree that these results were due solely to the antibiotic use reports is unclear. Antibiotic use reports align with the “tracking and reporting” core element of antibiotic stewardship. In addition, such reports could also contain clinical guidance on how to improve prescribing practices which would also comply with the core element of education and expertise. Antibiotic use reports have the advantage of directly addressing antimicrobial use rates without requiring the sort of proactive, direct intervention and time investment needed for audit and feedback. Antibiotic utilization reports may be less likely to lead to fewer antibiotic prescriptions if additional interventions are not in place.^20^ Antibiotic reports are a retrospective rather than prospective intervention and therefore are likely to require a longer period to show improvement in antibiotic prescribing practices. Creation of antibiotic use reports also are difficult without a common EHR and data tracking capability that may not be available to many rural settings. That said, such reports are well suited to the outpatient rural context as they can easily be disseminated electronically, less labor intensive than audit and feedback, and are scalable to larger patient populations.

### Peer comparison

Another variation of the audit and feedback approach is peer comparison. In such an intervention, individual providers receive a report comparing their antimicrobial prescribing practices to their peers, showing how frequently they prescribed antibiotics overall, how often antibiotics were prescribed in accordance with local guidelines, or other relevant metrics. Such reports would include comparison data for other providers in the program, clinic, or health system, allowing recipients of the reports to see how they benchmark.^14^ An example of such a report can be found in supplement 1. Peer comparison was a major component of a behavioral science-based stewardship intervention in the urgent care setting undertaken by Cummings et al in 2020; this intervention reduced inappropriate antibiotic prescriptions by 14.9 percentage points at three rural high-volume urgent care clinics.^21^ While this study was not specifically introduced as a tele-stewardship study, it illustrates the effectiveness of such an intervention. This approach has been used as a component of multiple successful stewardship interventions.^5,14^ An advantage of this approach is that providers may be incentivized to change their prescribing practices when they are engaged in friendly competition with their peers. Such interventions can also be generalized up to the clinic level, comparing locations instead of individuals, though this approach loses the advantages of individualized feedback. Drawbacks to peer comparison are that generating such individualized data is a time and labor-intensive process. It also may not be as applicable to very rural settings that do not have many comparator providers, though such situations would likely not be common.

### Education

Educational interventions are another common feature of tele-stewardship programs.^4,5,16,17,20,22^ These interventions focus on providing prescribers with up-to-date information regarding best practices associated with antimicrobial prescribing. Education could take a variety of forms including in person or virtual lectures or case-based discussions,^4,5,16,20,22^ written or electronic materials,^5,17,20^ and even internal podcasts.^5^ There are several advantages to educational interventions. Written materials and guides do not require an extensive time investment after their initial creation. Lecture or discussion-style interventions allow for direct engagement with prescribers. Successful educational interventions require fewer resources than audit and feedback. They are also more easily scaled to a large number of prescribers than audit and feedback, which is important when considering the rural outpatient context. There are limitations to educational interventions, however. The effectiveness of an educational intervention depends heavily on prescriber engagement. Lectures may not be attended by prescribers uninterested in antimicrobial stewardship, and written materials can be easily ignored by prescribers who feel their prescribing practices do not need to change. Strategies to increase provider engagement with antibiotic stewardship initiatives are needed, including offering continuing education credits, and requiring participation by clinic or health system leadership, emphasizing why leadership commitment was identified by the CDC as a pillar of any stewardship program.^6^ Stenehjem’s 2018 comparative study of different levels of stewardship intervention revealed that programs that included only interventions that required prescriber initiative to engage with such as provider education were less effective at reducing overall antibiotic use than programs that included strategies like audit and feedback where the antibiotic prescribing data is “pushed” to the providers by the stewardship team.^20^ Educational stewardship interventions have the potential for significant impact in rural settings because they are well suited to the unique challenges of such sites. This limitation has been seen in educational interventions ranging from antibiotic prescribing to depression treatment.^20,23^

### Local guideline creation

Creation of local guidelines regarding antimicrobial prescribing is a feature of several successful stewardship programs.^4,5,17,20^ Such programs have developed syndrome-specific local guidelines designed to support antimicrobial stewardship minded decision making. Most of these studies focused on implementing stewardship in small community hospitals, though one focused on urgent care clinics.^5^ These studies included adults and children across a variety of healthcare systems across the US. These interventions tend more toward action for policy and practice rather than education in terms of the CDC’s core elements. The advantages and disadvantages of local guidelines are similar to those associated with written educational interventions, however. Guideline creation has the additional advantage of carrying institutional approval- it is possible that providers will be more likely to follow stewardship recommendations when they know such recommendations have been approved by institutional authorities. This has the additional benefit of lending further credence to a provider’s recommendations when discussing antimicrobial choices with families, a benefit identified by Spencer et al in their 2022 qualitative assessment of pediatric antibiotic prescription decision making.^24^ The presence of local guidelines can facilitate further interventions such as implementation of clinical decision support tools into an EHR, as demonstrated by Khadem et al.^25^ The main adaptation necessary for outpatient rural implementation of this strategy would be to ensure that the guidelines covered conditions such as URIs and community acquired pneumonia where antimicrobial stewardship is most needed in outpatient medicine.

### Direct consultation with infectious disease experts

Another element sometimes included in tele-stewardship programs is direct consultation with ID experts, which is especially useful in rural areas that are often far from academic medical centers and lack access to subspecialists.^16,21,26^ This intervention would allow patients or providers to consult with specialists regarding questions related to antimicrobial prescribing. This would allow for prescribing advice to be tailored to each unique situation, an advantage over more general educational interventions. Such direct consultation has been reported by prescribers to be easy to use and supportive of patient care.^26^ Disadvantages include the need for prescribers to actively engage with the intervention for there to be any value added. Stenehjem’s 2018 study demonstrated no improvement in antibiotic use in interventions that included an ID hotline without more comprehensive audit and feedback interventions, though there was a decrease in the use of restricted antibiotics.^20^ This kind of intervention may be considered more of a telemedicine intervention rather than a tele-stewardship intervention considering its direct effect on clinical care, though it still bears mentioning as it is a common element bundled into tele-stewardship interventions and may help to increase provider “buy-in” to stewardship recommendations through relationship building with consultants.

### Electronic health record nudges

Electronic decision support at the time of order entry has been shown to decrease inappropriate antibiotic prescribing.^4,5,17,20,22^ These EHR nudges usually were implemented in inpatient settings with some studies including both children and adults. EHR changes included alteration of order sets, quick links to local guidelines or antibiograms, restricting antibiotics and requiring co-signatures by approving providers, and requiring further information prior to prescribing a particular antibiotic. These kinds of interventions generally require IT expertise at the beginning but then require little in terms of ongoing support making them easy to scale to larger populations. Restriction of certain antibiotics unsurprisingly leads to less use,^17,20^ although such interventions are unlikely to apply well to the outpatient rural context as the antibiotics restricted in hospitals are not used often in the outpatient context. Another limitation is that such interventions would also require a common EHR among the outpatient sites. Some rural sites may not use an EHR at all. Additionally, EHR modifications require substantial information technology (IT) resources and expertise, which may not always be available in all settings.

## Discussion and future research

A significant proportion of antibiotics^27^ are prescribed in rural outpatient clinics and these settings would benefit from antibiotic stewardship interventions. Optimal stewardship initiatives and implementation strategies for rural outpatient settings are not well-studied, but methods used in other settings may be effective.^1^ Several of the strategies examined in this review have not been investigated in the outpatient setting at all. Implementation studies investigating the effectiveness of different interventions over time would help design future interventions and focus resources on the strategies with the greatest impact (Table 3). It remains unclear to what degree current studies on inpatient tele-stewardship can be applied to the rural outpatient context. Future studies should evaluate comparative effectiveness of a variety of stewardship in rural outpatient primary care and urgent care clinics. Since outpatient locations differ in practice style, flow, and patient mix, interventions that are effective at primary care offices may not be similarly effective at urgent care sites. The presence or absence of a healthcare network is another factor that ought to be investigated as many interventions become much less effective without the presence of such a network. Future work in rural settings should also investigate inequities in antimicrobial prescribing practices and whether antibiotics are used differently based on patients’ race or other demographic characteristics. For example, Black children have been found to generally receive fewer antibiotics than non-Black children.^28^ Further implementation and evaluation of tele-stewardship strategies in outpatient rural settings is needed and likely to significantly reduce inappropriate antimicrobial prescriptions.

**Table 3.** Evidence supporting telehealth strategies in the inpatient and outpatient settings

Strategy	Has been part of an effective inpatient intervention	Has been part of an effective outpatient intervention
Audit and Feedback	Yes ^2,4,13,16,18,19,20^	Yes ^5,14,21^ (Primary and Urgent Care
Provider Education	Yes ^4,17,20,22^	Yes ^5,14,21^ (Primary and Urgent Care)
EHR Nudges	Yes ^4,17,20,22^	Yes ^5^ (Urgent Care)
Antibiotic Use Reports	Yes ^2,20^	Yes^14^ (Primary Care)
Consultation with ID providers	Yes^16^	No
Guideline creation	Yes ^4,22^	Yes^14^ (Primary Care)

## Supporting information

10.1017/ash.2025.10133.sm001 Peworchik et al. supplementary material Peworchik et al. supplementary material

## Supplementary material

For supplementary material accompanying this paper visit https://doi.org/10.1017/ash.2025.10133
