# Supplementary figures and images for: Antimicrobial tele-stewardship strategies and their application to the rural outpatient setting
Source: Antimicrob Steward Healthc Epidemiol. 2025 Oct 1;5(1):e240. doi: 10.1017/ash.2025.10133 (PMC12509144; doi:10.1017/ash.2025.10133)

Supplement: Example of Individualized Peer Comparison.


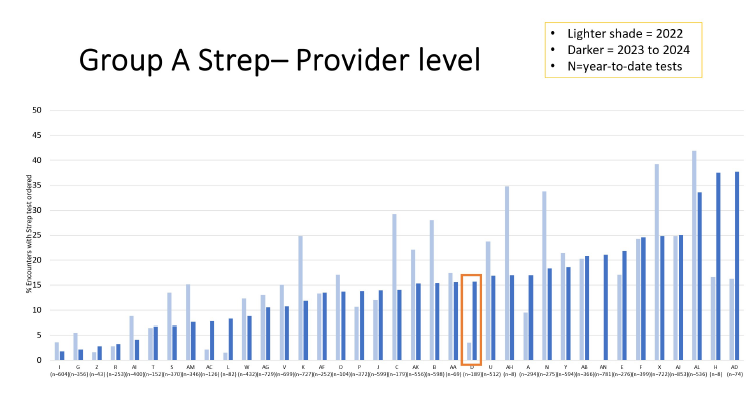

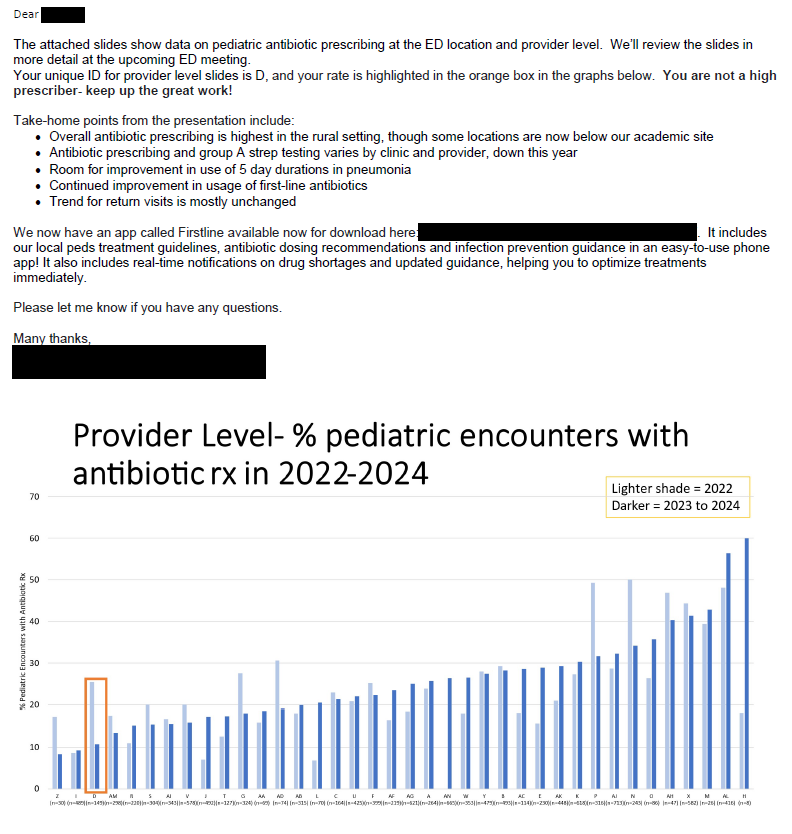

Supplement: Peworchik et al. supplementary material [file S2732494X25101332sup001.docx]
